# Eosinophil Activation by Toll-Like Receptor 4 Ligands Regulates Macrophage Polarization

**DOI:** 10.3389/fcell.2019.00329

**Published:** 2019-12-20

**Authors:** Jiyoung Yoon, Han-Na Um, Jinsun Jang, Young-An Bae, Woo-Jae Park, Hee Joo Kim, Mee-Sup Yoon, Il Yup Chung, YunJae Jung

**Affiliations:** ^1^Department of Microbiology, College of Medicine, Gachon University, Incheon, South Korea; ^2^Department of Health Sciences and Technology, GAIHST, Gachon University, Incheon, South Korea; ^3^Department of Dermatology, Gachon Gil Medical Center, College of Medicine, Gachon University, Incheon, South Korea; ^4^Department of Biochemistry, College of Medicine, Gachon University, Incheon, South Korea; ^5^Department of Molecular Medicine, College of Medicine, Gachon University, Incheon, South Korea; ^6^Department of Bionano Technology, Hanyang University, Ansan, South Korea

**Keywords:** eosinophils, EoL-1 cells, TLR4, THP-1 cells, macrophage polarization

## Abstract

Eosinophils are terminally differentiated granulocytes that have long been considered as destructive cells associated with Th2 type immune responses such as allergic inflammation and helminth infections. Recently, eosinophils have been actively studied as multifunctional leukocytes regulating an array of physiological responses through interaction with other immune cells. In this study, we examined the expression and function of Toll-like receptors (TLRs) in eosinophilic EoL-1 cells and demonstrated the expression of a number of immune mediators in activated EoL-1 cells and their interaction with the macrophage cell line THP-1 upon TLR4 ligand stimulation. EoL-1 cells differentiated with butyrate increased expression of TLR3, TLR4, and TLR7 at mRNA and protein level with flow cytometry analysis. Mature eosinophils derived from human cord blood CD34^+^ cells were subjected to RNA-sequencing, and showed the expression of a panel of TLR transcripts and TLR4 was the most highly expressed TLR. Among the cognate ligands of TLR3, TLR4, and TLR7, lipopolysaccharide (LPS) or palmitic acid significantly increased mRNA expression of immune mediators in differentiated EoL-1 cells. Notably, Western blot analysis of palmitic acid-treated differentiated EoL-1 cells showed significantly up-regulated expression of Th2 type cytokines and transcription factors driving eosinophil differentiation. To evaluate functional significance of TLR4 ligand-stimulated eosinophils, we added conditioned media (CM) from EoL-1 cells to differentiated THP-1 cells and assessed the expression of M1 macrophage or M2 macrophage-related markers. M1 and M2 macrophage markers were significantly upregulated by CM from LPS and palmitic acid stimulated EoL-1 cells, respectively. In addition, the adipose tissue of obese mice, where eosinophils are decreased due to obesity-induced inflammation, showed significantly decreased frequency of M2 macrophages, despite an increase in the total macrophage numbers. Based on these collective data, we proposed that eosinophils regulate both inflammatory and anti-inflammatory polarization of macrophages through functional changes induced by different TLR4 ligands.

## Introduction

Eosinophils are terminally differentiated innate immune cells, classically described as being involved in allergic diseases and helminth parasitic infections (Gleich and Adolphson, 1986; Rothenberg and Hogan, 2006). The cytoplasm of eosinophils contains secondary toxic granules such as eosinophil peroxidase, eosinophil cationic protein, eosinophil-derived neurotoxin, and major basic proteins, and the granules also store numerous cytokines, enzymes, and growth factors (Rosenberg et al., 2013). Eosinophils are generally thought to be pro-inflammatory cells because the release of cytotoxic granule proteins from eosinophils causes epithelial damage, smooth muscle constriction, and recruitment of inflammatory cells (Rothenberg et al., 2001; Jung and Rothenberg, 2014). However, increasing evidence supports that eosinophils are multifunctional leukocytes regulating various physiologic responses; eosinophils support tissue regeneration (Heredia et al., 2013), promote homeostatic intestinal immune responses (Jung et al., 2015), regulate adipose tissue macrophage polarization and glucose metabolism (Wu et al., 2011; Lee et al., 2018), and mediate the killing of bacteria, viruses, and fungi (Inoue et al., 2005; Dyer et al., 2009; Linch et al., 2009; Qiu et al., 2014). Therefore, it is necessary to identify the underlying mechanism that supports the multifunctional aspects of eosinophils as immune-modulatory cells.

Toll-like receptors (TLRs) are molecular regulators through which the immune system may sense the environment as TLRs recognize molecules broadly shared by pathogens (pathogen-associated molecular patterns) or endogenous molecules derived from damaged tissues (damage-associated molecular patterns) (Akira et al., 2006; Kumar and Bot, 2018). Although the expression of TLRs in eosinophils is relatively low compared to other granulocytes such as neutrophils (Nagase et al., 2003), all TLRs except TLR8 are expressed in the eosinophils (Kvarnhammar and Cardell, 2012). TLR7 is known to be the most prominent TLR in eosinophils, as supported by TLR7-induced eosinophil activation in asthma (Hiraguchi et al., 2011), and activation of neutrophil upon TLR7 ligation in the eosinophilic cell line EoL-1 (Kim et al., 2018). However, the precise expression profile and functions of TLRs in eosinophils have remained largely unknown, especially in modulating immune responses by interacting with other immune cells.

Macrophages are plastic cells that respond and adapt to tissue microenvironments through phenotypic differentiation (Henze and Mazzone, 2016). Based on pathological or physiological stimuli, the circulating monocytes can be differentiated into two types of macrophages (Huh et al., 2014). Macrophages can exhibit either pro- or anti-inflammatory phenotypes and are routinely classified into the M1 and M2 phenotypes (Lichtnekert et al., 2013). Macrophages activated by interferon (IFN)-γ or pathogen-associated molecular patterns, such as lipopolysaccharide (LPS), are polarized to the M1 phenotype and produce high levels of proinflammatory cytokines, reactive oxygen, and nitrogen species that drive microbicidal activities (Lichtnekert et al., 2013; Tan et al., 2015). In contrast, macrophages differentiated with Th2 cytokines or immune regulating cytokines, such as transforming growth factor-β are polarized to the M2 macrophages that inhibit inflammation, promote tissue repair, and support homeostatic responses (Shapouri-Moghaddam et al., 2018). In addition, distinction between M1 and M2 macrophage polarization has significant roles in adipose tissue inflammation and glucose metabolism in obesity (Castoldi et al., 2015). Lean adipose tissue is abundant in eosinophils, Th2 cells, and M2 macrophages that secrete Th2 cytokines and IL-10, and promote adipose tissue homeostasis and insulin sensitivity (Castoldi et al., 2015). In the obese state, the neutrophils, Th1 lymphocytes, and M1 macrophages are infiltrated in the adipose tissue and actively secrete pro-inflammatory cytokines that activate pro-inflammatory signaling and insulin resistance (Castoldi et al., 2015). A recent study has uncovered that eosinophils maintain the adipose tissue M2 macrophages as major contributors of Th2 cytokines (Wu et al., 2011; Lee et al., 2018); however, the mechanism underlying the interaction between eosinophils and macrophages is still incompletely understood.

In this study, we assessed the expression and function of TLRs in differentiated eosinophilic EoL-1 cells (dEoL-1) and mature eosinophils differentiated from human cord blood (CB) CD34^+^ cells. We demonstrated the functional expression of TLR4 in EoL-1 cells and showed that ligation of TLR4 on eosinophils with LPS or palmitic acid activates eosinophils, which further induce macrophage polarization toward M1 or M2, respectively. In addition, we showed that M2 macrophages are significantly decreased in the adipose tissue of obese mice with a decrease in eosinophils, despite an increase in the total and M1 macrophages. Collectively, we propose that eosinophils regulate macrophage polarization through their functional immune response in response to different TLR4 ligands.

## Materials and Methods

### Cell Culture

EoL-1 cells were maintained at 1 × 10^6^/mL in Roswell Park Memorial Institute (RPMI) 1640 medium (Sigma–Aldrich, St. Louis, MO, United States) supplemented with 10% fetal bovine serum in 5% CO_2_ at 37°C and differentiated by adding 0.5 μM butyrate (Sigma–Aldrich, St. Louis, MO, United States) for 2 days. To examine the effects of TLR ligands on the differentiated EoL-1 cells, butyrate-treated EoL-1 cells were stimulated with poly(I:C) (10 μM, InvivoGen, San Diego, CA, United States), LPS (1 ng/mL, Sigma–Aldrich, St. Louis, MO, United States), palmitic acid (250 mM, Sigma–Aldrich, St. Louis, MO, United States), or R848 (1 μg/mL, InvivoGen, San Diego, CA, United States) for 24 h. Human eosinophils were differentiated from CB CD34^+^ cells for 24 days as previously described in detail (Hwang et al., 2016, 2018).

### Real-Time PCR Analysis

Total RNA was extracted using the QIAzol lysis reagent (Qiagen, Hilden, Germany) and subsequently column-purified with an RNeasy Mini Kit (Qiagen, Hilden, Germany). RNA (500 ng) was treated with DNase I (New England Biolabs, Ipswich, MA, United States), and cDNA was synthesized using an iScript cDNA synthesis kit (Bio-Rad Laboratories, Hercules, CA, United States). Real-time PCR was performed using iQ SYBR Green Supermix (Bio-Rad Laboratories, Hercules, CA, United States) on a CFX Connect real-time PCR detection system (Bio-Rad Laboratories, Hercules, CA, United States). The primers are detailed in Supplementary Table S1.

### RNA-Sequencing of CB-Differentiated Eosinophils

cDNA library was prepared for RNA-sequencing and data analysis was performed as previously described (Hwang et al., 2016). Total RNA was extracted from CB eosinophils on day 24 using TRIzol reagent, and ribosomal RNA was removed from total RNA using the RNAMius Transcriptome Isolation kit (Life Technologies, Grand Island, NY, United States). Using 100 ng of extracted RNA, RNA libraries were then generated using the NEBNext Ultra Directional RNA Library Prep Kit from Illumina (San Diego, CA, United States). The cDNA fragments were sequenced in paired end lanes using the Illumina HiSeq2500 (National Instrumentation Center for Environmental Management in Seoul National University, Seoul, Korea). RNA-sequencing was performed on three independent RNA samples from each of the three donors. For the analysis of differentially expressed genes, the quality-checked reads for each sample were processed using TopHat (version 2.0.10) software based on the *Homo sapiens* UCSC hg19 reference genome sequence. The gene-expression values for each sample were calculated by Cufflinks 2 based on the fragment per kilobase per million map reads (FPKM) method and a FPKM > 1 of the analyzed samples was used to filter for potentially significant expression of TLR.

### Flow Cytometry

To characterize TLR expression, 1 × 10^6^ differentiated EoL-1 cells were resuspended in 100 μl of phosphate-buffered saline (PBS) containing 10% fetal calf serum, 20 mM 4-(2-hydroxyethyl)-1-piperazineethanesulfonic acid, and 10 mM ethylenediaminetetraacetic acid. After blocking Fc receptors with Human TruStain FcX (BioLegend, San Diego, CA, United States) for 15 min at 4°C, the cells were stained for 30 min at 4°C with the following antibodies: Anti-TLR4 (HTA125) and TLR9 (eB72-1665) from BioLegend, San Diego, CA, United States. Intracellular expression of TLR3 or TLR7 was determined by staining with anti-TLR3 (TLR3.7, BioLegend, San Diego, CA, United States) or TLR7 (4G6, ThermoFisher Scientific, Waltham, MA, United States) following fixation and permeabilization using a Cytofix/Cytoperm Kit (BD Biosciences, San Diego, CA, United States). To characterize the surface phenotype of cells isolated from murine adipose tissues, 1 × 10^6^ cells were stained for 30 min at 4°C with the following antibodies after blocking Fc receptors with anti-mouse CD16/CD32 (2.4G2, BD Biosciences, San Diego, CA, United States): mAb against CCR3 (83101) from R&D Systems (Minneapolis, MN, United States); anti-SiglecF (E50-2440) and anti-CD11c (HL3) from BD Biosciences, San Diego, CA, United States; anti-CD45 (30-F11), F4/80 (BM8), and CD206 (141708) from BioLegend, San Diego, CA, United States. Except for anti-TLR4 (1:20), all antibodies were diluted 1:100. Each sample was analyzed with a FACSCalibur flow cytometer (BD Biosciences, San Diego, CA, United States) and the data were processed using FlowJo software (Tree Star, Ashland, OR, United States).

### Protein Array Analysis of Cytokines in Culture Supernatant of EoL-1 Cells

Culture supernatants were collected from butyrate-treated, butyrate and LPS-treated, and butyrate and palmitic acid-treated EoL-1 cells. The expression profile of 36 different cytokines in 1 mL of each sample was assessed using a Human Cytokine Array kit using the instructions provided by the manufacturer (R&D Systems, Minneapolis, MN, United States). Chemiluminescence signals produced in proportion to the amount of cytokines were detected using a biomolecular imager (ImageQuant LAS 4000, GE Healthcare Bio-Sciences AB, Uppsala, Sweden).

### Total Protein Extraction and Western Blot Analysis

The cell lysates were prepared in ice-cold tissue lysis RIPA buffer (ThermoFisher Scientific, Waltham, MA, United States) containing a protease inhibitor cocktail (ThermoFisher Scientific, Waltham, MA, United States) and phosphatase inhibitor cocktail (ThermoFisher Scientific, Waltham, MA, United States), and the total protein was extracted. Samples from tissue lysates were resolved by SDS–PAGE and then transferred to a nitrocellulose membrane. After 1 h blocking at 4°C using 5% bovine serum albumin (BSA) in PBS containing 0.1% Tween-20, the membrane was incubated overnight with antibodies against phospho-p38 MAP kinases (MAPK), p38 MAPK, phospho-p44/42 MAPK, p44/42 MAPK, GATA binding protein 1 (GATA-1) and GATA-3 purchased from Cell Signaling Technology (Danvers, MA, United States), and β-actin (Novusbio, Centennial, CO, United States) at 1:1000 dilution in PBS containing 0.1% Tween-20 at 4°C. Except for anti-β-actin (30 μg), all antibodies were allowed to react with 50 μg of extracted protein. After three PBST washes, membranes were incubated with a secondary antibody for 1 h at room temperature. Chemiluminescence was performed using a Pierce ECL Western Blotting Substrate (ThermoFisher Scientific, Waltham, MA, United States).

### THP-1 Polarization

Human monocyte THP-1 cell line was maintained at 1 × 10^6^/mL and stimulated with 100 ng/mL of phorbol 12-myristate 13-acetate (PMA) (Sigma–Aldrich, St. Louis, MO, United States) for 24 h for M0 differentiation. M0 differentiated THP-1 cells were further stimulated with conditioned media (CM) collected from butyrate-differentiated, butyrate-differentiated/LPS-treated, or butyrate-differentiated/palmitic acid-treated EoL-1 cells to induce polarization.

### High Fat Diet Feeding and Preparation of Cell Suspension of White Adipose Tissues

C57BL/6 female mice (Orientbio, Gyeonggi, South Korea) were housed under standard temperature and humidity in the specific pathogen-free facilities of Gachon University. Eight-to-ten-week-old mice were fed a high fat diet (HFD) (60% fat, D12492, Research Diets, New Brunswick, NJ, United States) or a chow diet (5.0% fat, 5053, LabDiet, St. Louis, MO, United States) for 12 weeks (*n* = 3 mice/group). Animal procedures were reviewed and approved by the Center of Animal Care and Use of Lee Gil Ya Cancer and Diabetes Institute, Gachon University (Number: LCDI-2018-0062). At sacrifice, all the mice were weighed and the visceral fat was removed and weighed. Visceral adipose tissue or small intestine was incubated with 0.2% collagenase II (Sigma–Aldrich, St. Louis, MO, United States) in RPMI 1640 medium/0.5% BSA with continuous stirring at 37°C for 30 min. The isolated cells were filtered through a 100 μM cell strainer and red blood cells were lysed. For the enrichment of leukocytes, the cells were subjected to density-gradient centrifugation in 40/75% Percoll (Sigma–Aldrich, St. Louis, MO, United States). The cells harvested from the interface were washed and used in subsequent assays.

### Statistical Analysis

Two-group comparisons were performed with either an unpaired Student’s *t*-test or Mann–Whitney *U*-test. Data differences between groups were examined for statistical significance using one-way ANOVA with the Tukey’s *post hoc* test or Kruskal–Wallis test with the Dunn’s *post hoc* test. The normality of data distribution was analyzed using the Kolmogorov–Smirnov test. A *p*-value < 0.05 was considered significant. GraphPad Prism 5 (GraphPad, San Diego, CA, United States) was used for data analysis. All data values are presented as mean of replicates ± standard deviation (SD).

## Results

### TLR Expression Is Induced in Differentiated EoL-1 Cells and Eosinophils Derived From CB CD34^+^ Cells

**FIGURE 1:**
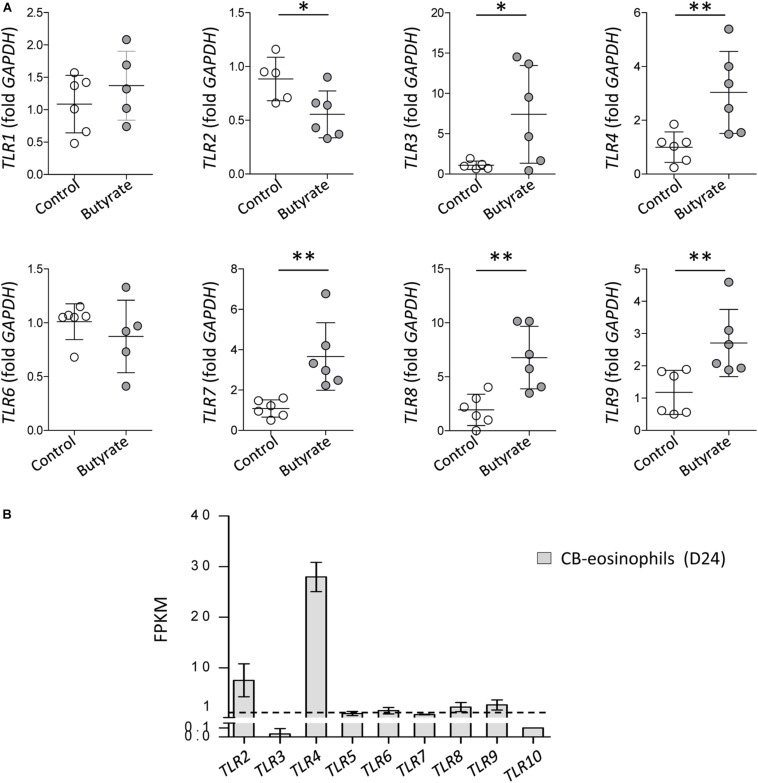
Toll-like receptors (TLRs) expression in EoL-1 cells and mature eosinophils differentiated from human cord blood (CB) CD34^+^ cells. **(A)** mRNA expression of TLRs in butyrate-differentiated EoL-1 (dEoL-1) cells. The mRNA expression for *TLR1*, *TLR2*, *TLR3*, *TLR4*, *TLR6*, *TLR7*, *TLR8*, and *TLR9* in undifferentiated EoL-1 cells and dEoL-1 cells was analyzed using real-time PCR. Data are represented as mean ± SD. ^∗^*p* < 0.05, ^∗∗^*p* < 0.01 (Student’s *t*-test for *TLR1*, *TLR2*, *TLR3*, *TLR4*, *TLR7*, *TLR8*, and *TLR9*, Mann–Whitney *U*-test for *TLR6*). **(B)** Expression of TLR genes in CB-differentiated eosinophils. The expression was analyzed by RNA-sequencing and the values represent fragment per kilobase per million map reads (FPKM) of genes. Dashed line indicates FPKM = 1. (*n* = 3/group). Data are represented as mean ± SD.

**FIGURE 2:**
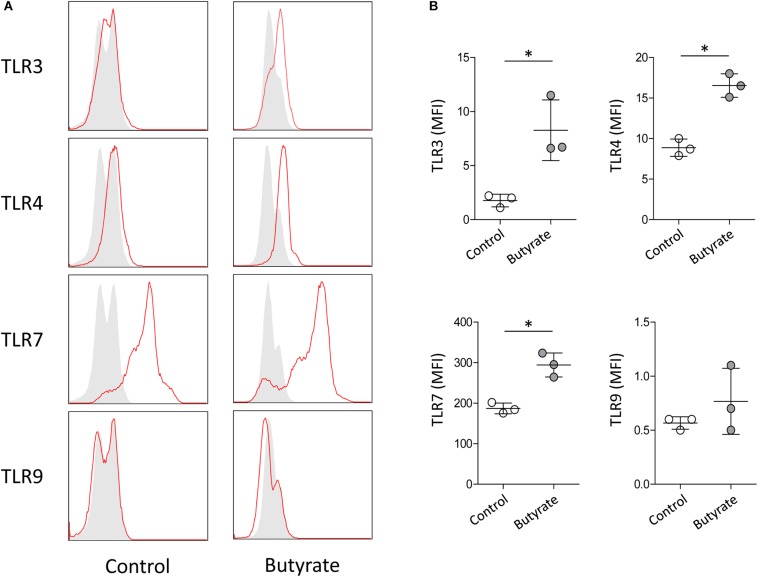
Protein expression level of TLR3, TLR4, TLR7, and TLR9 in dEoL-1 cells. **(A)** Representative flow cytometry analysis of intracellularly expressed TLR3 and TLR7 and surface-expressed TLR4 and TLR9 in undifferentiated EoL-1 cells (control) and dEoL-1 cells. Isotype controls are in gray. **(B)** Mean fluorescence intensity (MFI) of TLR3, TLR4, TLR7, and TLR9 of indicated EoL-1 cells is shown (after subtraction of isotype control MFI). Data are representative of two independent experiments. Data are represented as mean ± SD. ^∗^*p* < 0.05 (Mann–Whitney *U*-test).

Purification of eosinophils from peripheral blood is difficult because eosinophils account for <5% of circulating leukocytes in normal humans and have a short life span (Wong et al., 1999). A human eosinophilic cell line EoL-1 represents a useful model for characterizing eosinophils and EoL-1 cells can be differentiated into mature eosinophil-like cells by butyrate treatment (Jung, 2015). In accordance with previous reports, expression of mature eosinophil marker *CCR3*, eosinophil secondary granules (*ECP* and *EDN*), and IL-5Rα was significantly increased in dEoL-1 cells treated with butyrate (Supplementary Figure S1). We next checked expression levels of TLRs in EoL-1 cells and observed that transcripts of TLR3, TLR4, TLR7, TLR8, and TLR9 were significantly increased in dEoL-1 cells (Figure 1A). Eosinophils arise from CD34^+^ hematopoietic stem cells (Rothenberg and Hogan, 2006), and we previously demonstrated that culturing CB CD34^+^ cells for 24 days with eosinophil lineage committed cytokines produces mature eosinophils (Hwang et al., 2016, 2018; Supplementary Figure S2). As shown in Figure 1B, we found apparent mRNA expression for TLR2, TLR4, TLR6, TLR8, and TLR9 in CB-derived eosinophils (FPKM > 1), and expression of TLR4 transcript was the highest compared to other TLRs (FPKM = 27.97 ± 1.67). Flow cytometry analysis demonstrated significant increase of surface TLR4, and intracellular TLR3 and TLR7 in dEoL-1 cells compared to undifferentiated EoL-1 cells (Figure 2).

### Inflammatory Cytokine Expression Is Increased in dEoL-1 Cells With TLR4 Ligand Stimulation

**FIGURE 3:**
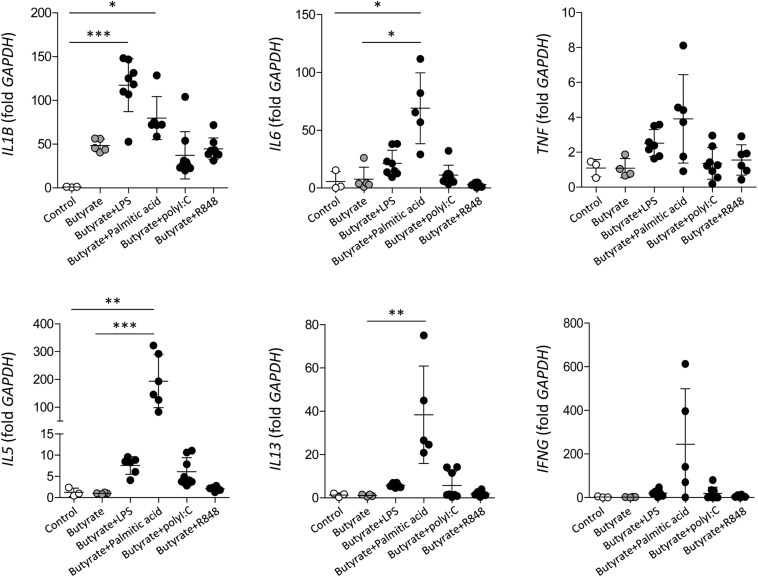
Inflammatory cytokine production in dEoL-1 cells with TLR ligand stimulation. mRNA expression of *IL1B*, *IL6*, *TNF*, *IL5*, *IL13*, and *IFNG* in indicated EoL-1 cells was analyzed by real-time PCR. Data are represented as mean ± SD. *^∗^p* < 0.05, ^∗∗^*p* < 0.01, *^∗∗∗^p* < 0.001 (Kruskal–Wallis test).

To evaluate whether significantly increased TLR3, TLR4, and TLR7 expression in dEoL-1 cells has a functional significance, we treated dEoL-1 cells with respective ligands of these TLRs, and examined induction of inflammatory cytokines. Functionally distinctive ligands are available for TLR4 activation; LPS, which is the major component of the cell wall of Gram-negative bacteria (Sabroe et al., 2002), and palmitic acid, the representative saturated fatty acid abundant in the adipose tissue (Mittendorfer, 2011). Therefore, dEoL-1 cells were treated with different concentrations of LPS or palmitic acid, and 1 ng/mL of LPS and 250 μM of palmitic acid were chosen based on their ability of inducing highest levels of examined cytokine transcript (Supplementary Figure S3). As shown in Figure 3, mRNA expression of *IL1B*, *IL6*, *IL5*, and *IL13* was significantly higher in dEoL-1 cells treated with either LPS or palmitic acid compared to poly I:C (ligand of TLR3) or R848 (ligand of TLR7)-treated dEoL-1 cells. The expression of Th2 cytokine transcripts (*IL5* and *IL13*) was significantly increased in the dEoL-1 cells treated with palmitic acid (Figure 3). Although butyrate increased eosinophil-lineage markers in EoL-1 cells (Supplementary Figure S1), expression of inflammatory cytokines in dEoL-1 cells was not significantly upregulated compared to undifferentiated cells (Figure 3). In conclusion, stimulating eosinophils with TLR4 ligands induces upregulation of immune response regulating cytokines.

### Cytokine Secretion Profiles of dEoL-1 Cells Induced by LPS or Palmitic Acid Are Functionally Distinguishable

**FIGURE 4:**
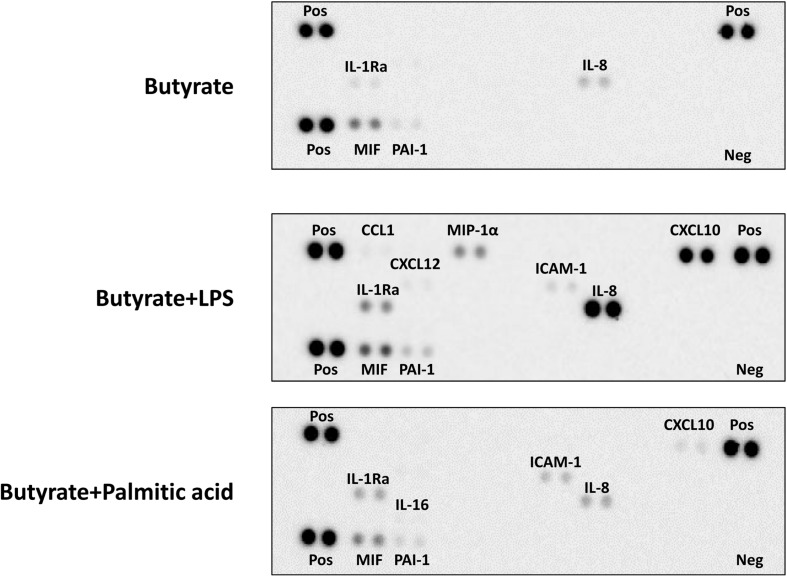
Secretion of immune mediators in dEoL-1 cells with TLR4 ligands stimulation. Thirty-six different cytokines in the culture supernatants of butyrate, butyrate/LPS, or butyrate/palmitic acid-stimulated EoL-1 cells were semi-quantitated using a human cytokine array. Quantification was performed with pooled culture supernatants collected from two independent experiments. Pos, positive control; Neg, negative control.

To extensively screen the inflammatory mediator producing ability of TLR4-stimulated dEoL-1 cells, the culture supernatants of butyrate-treated, butyrate and LPS-treated, and butyrate and palmitic acid-treated EoL-1 cells were semi-quantified using a human cytokine array. As shown in Figure 4, Supplementary Figure S4, and Supplementary Image S1–S3, the secretion of CCL1, CXCL10, and CXCL12, IL-8, ICAM-1, MIP-1α, MIF, and PAI-1, which are involved in inflammatory activation (Gombert et al., 2005; Lawson and Wolf, 2009; Takahashi et al., 2009; Cesari et al., 2010; Karin, 2010; Zhang et al., 2014; Kim et al., 2018), was increased in the dEoL-1 cells treated with LPS. Although palmitic acid-treated dEoL-1 cells showed increased production of CXCL10 and ICAM-1 than the dEoL-1 cells, the CXCL10 expression level was lower than that of LPS-treated cells, and the expression of IL-8, MIF, and PAI-1 was not different compared to the dEoL-1 cells (Figure 4, Supplementary Figure S4, and Supplementary Image S1–S3). The production of IL-16, which mediates immune regulation and eosinophil activation (Cruikshank et al., 2000; Ghigo et al., 2010; Luna-Gomes et al., 2013), was detected only in the palmitic acid-treated dEoL-1 cells. Collectively, these findings indicated that stimulation of the dEoL-1 cells with cognate ligands to TLR4 induces cytokine secretion from dEoL-1 cells, and the cytokine profile induced by LPS stimulation shifts to pro-inflammatory properties compared to palmitic acid stimulation.

### TLR4 Ligands Stimulation Changed Expression of MAPK Signaling Molecules, GATA-1, and GATA-3 in dEoL-1 Cells

**FIGURE 5:**
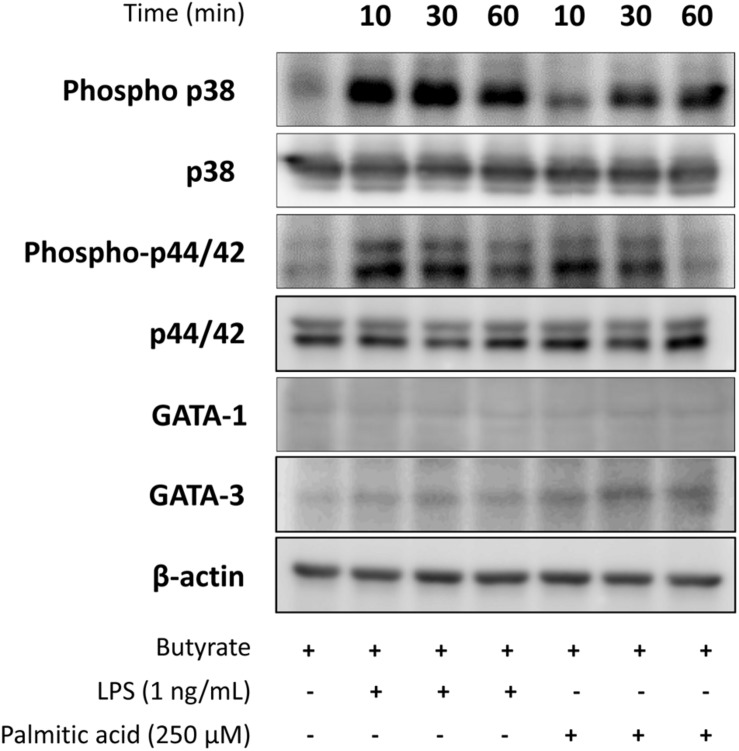
Expression of mitogen-activated protein kinases (MAPK) signaling molecules, and GATA-1 and GATA-3 transcription factor in dEoL-1 cells stimulated with TLR4 ligands. Differentiated EoL-1 cells were treated with LPS (1 ng/mL) or palmitic acid (250 mM) for 10, 30, or 60 min, lysates were prepared and expression of MAPK signaling molecules, GATA-1, and GATA-3 levels was determined by Western blot. Two independent experiments were performed and representative results are shown.

To further dissect the molecular mechanisms behind distinctive cytokine production induced by LPS or palmitic acid in dEoL-1 cells, we examined expression of MAPK signaling molecules, which activate in response to TLR4 stimulation (Rao, 2001; Prince et al., 2011), and eosinophil-lineage associated transcription factors. As MAPK signaling molecules comprise the largest class of phosphatase (Theodosiou and Ashworth, 2002), detection of phosphatase activity of phosphorylated p38 and p44/42 MAPK could be inhibited by the protease and phosphatase inhibitor cocktail added during protein extraction from dEoL-1 cells, although we used the inhibitors under conditions recommended for blocking the uncontrolled activity of phosphatases and proteinase. However, both LPS and palmitic acid induced phosphorylation of p38 and p44/42 MAPK in dEoL-1 cells (Figure 5 and Supplementary Image S4–S10). Phosphorylation of p38 was induced earlier and to a greater extent by LPS than palmitic acid, and p44/42 phosphorylation lasted longer and was higher in LPS-treated dEoL-1 cells than palmitic acid treated group (Figure 5 and Supplementary Image S4–S10). On the other hand, expression of GATA-1 and GATA-3, transcription factors associated with eosinophil differentiation and IL-5 expression (Yamaguchi et al., 1998; Zhang et al., 1998), was increased in dEoL-1 cells stimulated with palmitic acid (Figure 5 and Supplementary Image S4–S10). Taken together, stimulating dEoL-1 cells with LPS promoted strong MAPK signaling, whereas palmitic acid supported maturation of dEoL-1 cells into eosinophil-lineage development.

### CM From TLR4 Ligand-Treated dEoL-1 Cells Modulates Macrophage Polarization Induced in THP-1 Cells

**FIGURE 6:**
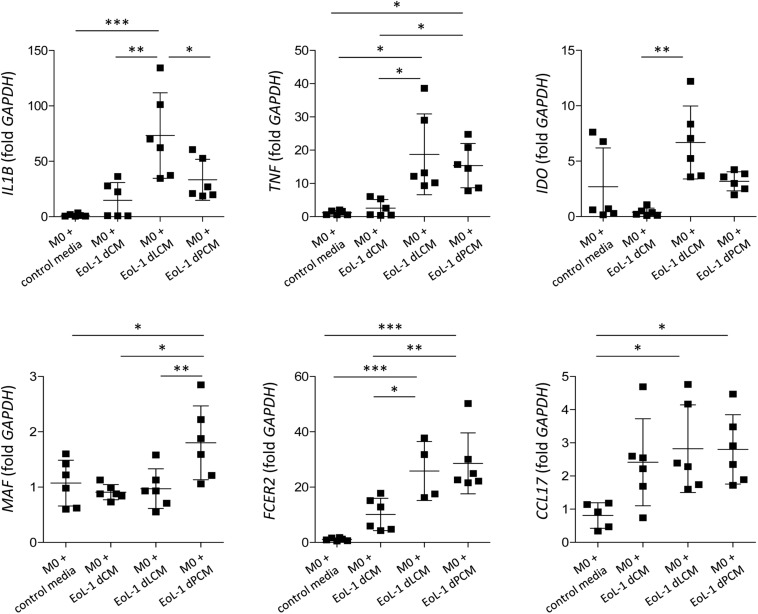
Polarization of M0-differentiated THP-1 cell by conditioned media (CM) from dEoL-1 cells stimulated with TLR4 ligands. M0-differentiated THP-1 cells were stimulated with CM collected from butyrate-differentiated EoL-1 cells (EoL-1 dCM), butyrate-differentiated/LPS-stimulated (EoL-1 dLCM), or butyrate-differentiated/palmitic acid-stimulated EoL-1 cells (EoL-1 dPCM) for 24 h. Expression of M1- (*IL1B*, *TNF*, and *IDO*) or M2-representing markers (*MAF*, *FCER2*, and *CCL17*) in M0- or CM-treated M0 THP-1 cells was analyzed by real-time PCR. Data are represented as mean ± SD. *^∗^p* < 0.05, ^∗∗^*p* < 0.01, *^∗∗∗^p* < 0.001 (one-way ANOVA for *IL1B*, *MAF*, *FCER2*, and *CCL17*, Kruskal–Wallis test for *TNF* and *IDO*).

Eosinophils are known to regulate macrophage polarization, and adipose tissue eosinophils are critical for maintaining M2 macrophages (Wu et al., 2011). To evaluate detailed interaction between eosinophils and macrophages, we cultured THP-1 cells with CM collected from LPS- or palmitic acid-treated dEoL-1 cells. M1 macrophages are characterized by the expression of inflammatory cytokines, such as IL-1β and tumor necrosis factor (TNF)-α, and expression of IFN-γ-inducible indoleamine 2.3-dioxygenase (IDO), based on their production of high levels of pro-inflammatory cytokines (Wang et al., 2014; Orecchioni et al., 2019). In contrast, macrophage-activating factor (MAF), CD23 (a low affinity IgE receptor), and CCL17 are proposed as markers of M2 activation, as M2 macrophages are characterized by their involvement in immune regulation and tissue remodeling (Roszer, 2015). As shown in Supplementary Figure S5, PMA-treated M0 THP-1 cells can be polarized to M1 or M2 phenotype by addition of LPS/IFN-γ or IL-4/IL-13, respectively. We further cultured M0 THP-1 cells with CM collected from dEoL-1 (dCM), LPS-treated dEoL-1 (dLCM), or palmitic acid-treated dEoL-1 cells (dPCM) for 24 h. M0 THP-1 cells cultured in dLCM showed significantly increased expression of *IL1B*, *TNF*, and *IDO*, markers representing M1 phenotype than M0 THP-1 or dCM-treated M0 THP cells (Figure 6). The expression of M2 phenotype markers *MAF* and *FCER2* (gene encoding CD23), and *TNF* was significantly upregulated in M0 THP-1 cells cultured in dPCM than M0 THP-1 and dCM-treated M0 THP cells (Figure 6). M0 THP cells cultured in dLCM and dPCM showed significantly increased expression of *IL1B* and *MAF*, respectively (Figure 6). These findings suggest that LPS stimulated eosinophils secrete factors that support M1 polarization and palmitic acid promotes eosinophils to support M2 polarization, and that microenvironment signals sensed by TLR4 in eosinophils modulate both inflammatory and anti-inflammatory immune responses.

### Adipose Tissue of HFD-Fed Mice Showed Decreased Eosinophils and M2 Macrophages

**FIGURE 7:**
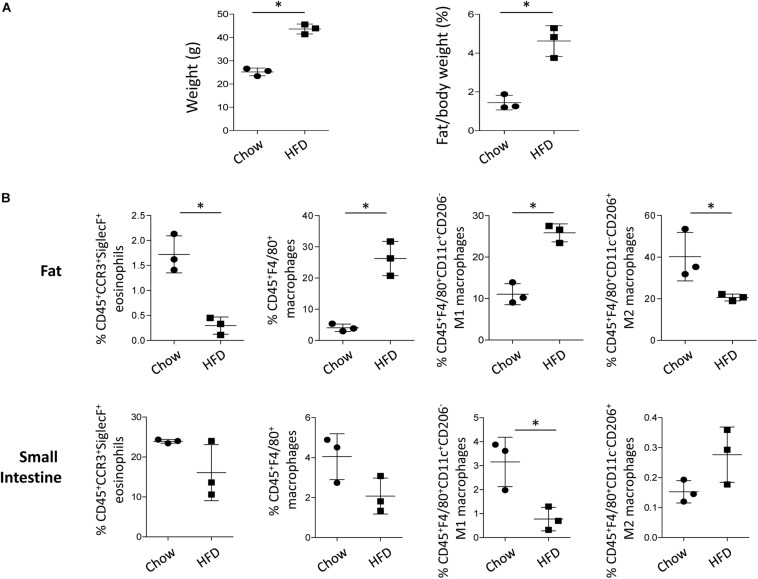
Changes in body weight and cell frequency in the adipose tissue and small intestine of mice on a high fat diet (HFD). **(A)** Body weight and fat/body mass of mice on a HFD (*n* = 3 mice/group). Data are mean ± SD. ^∗^*p* < 0.05 (Mann–Whitney *U*-test). **(B)** Flow cytometry analysis of eosinophils, macrophages, M1 macrophages, and M2 macrophages in the adipose tissue and small intestine of mice subjected to HFD. CD45^+^CCR3^+^SiglecF^+^ cells were gated for eosinophils and CD45^+^F4/80^+^ cells were gated for macrophages. Based on CD11c or CD206 expression, macrophages were further characterized into M1 (CD45^+^F4/80^+^CD11c^+^CD206^–^) and M2 subsets (CD45^+^F4/80^+^CD11c^–^CD206^+^). Data are represented as mean ± SD. ^∗^*p* < 0.05 (Mann–Whitney *U*-test).

Eosinophils are residential cells in the visceral adipose tissue, where a large amount of fatty acid is stored under homeostatic conditions (Berry, 1997). As eosinophils are closely associated with the maintenance of M2 macrophages in the adipose tissue (Wu et al., 2011), we checked the frequency of the eosinophils and polarized macrophages in the white adipose tissue of mice on a HFD that induces a significant increase in weight and fat mass (Figure 7A). The frequency of eosinophils in the lean adipose tissue was 1.7 ± 0.4%, whereas the frequency was only 0.3 ± 0.2% in the obese adipose tissues (Figure 7B and Supplementary Figure S6A). The frequency of M2 macrophages also significantly decreased in the adipose tissue of obese mice with a decrease in eosinophils, despite an increase in the total and M1 macrophages (Figure 7B and Supplementary Figure S6B). Under physiological conditions, eosinophils constitute major immune cell population in the lamina propria of the small intestine (Jung and Rothenberg, 2014; Jung et al., 2015), and as shown in Figure 7B and Supplementary Figure S6, the frequency of small intestinal eosinophils was not significantly different between mice fed the chow diet and HFD. The frequency of the total and M2 macrophages in the small intestine was also not significantly changed with a HFD (Figure 7B and Supplementary Figure S6B). The viability of cells isolated from the adipose tissue and small intestine was not significantly different between mice fed the chow and HFD (Supplementary Figure S7).

## Discussion

Eosinophils have long been considered as destructive cells associated with type 2 immune responses, including allergic inflammation and helminth infections (Rothenberg and Hogan, 2006). However, accumulating evidence has indicated the multifunctional role of eosinophils as modulators of immune responses (Jung and Rothenberg, 2014; Wen and Rothenberg, 2016). Eosinophils serve as a source of a variety of cytokines, growth factors, and lipid mediators, and express several pattern recognition receptors, including TLRs, nucleotide-binding oligomerization domains, and receptors for advanced glycation end products (Kvarnhammar and Cardell, 2012; Weller and Spencer, 2017).

We examined EoL-1 cells and showed a significant increase of TLR3, TLR4, and TLR7 at both mRNA and protein level by butyrate-induced differentiation (Figures 1, 2). Expression of a panel of TLRs was also observed in mature eosinophils derived from human CB and TLR4 transcript was the most abundant among the TLRs (Figure 1). LPS is one of the best studied immunostimulatory components of bacteria and is known to induce activation of dendritic cells, neutrophils, and macrophages through TLR4 signaling (Lu et al., 2008). LPS-induced secretion of inflammatory cytokines and secondary toxic granules was also reported in human eosinophils (Takanaski et al., 1994; Plotz et al., 2001). In line with these observations, treating dEoL-1 cells with LPS induced significant upregulation of an array of inflammatory mediators associated with various immune responses (Figures 3, 4). In addition to LPS, palmitic acid, a long chain saturated fatty acid, activates immune cells through TLR4 stimulation (Nicholas et al., 2017), and we could observe increased expression of *IL6*, *TNF*, and *IFNG* and secretion of CXCL10 and ICAM-1 in the dEoL-1 cells upon stimulation with palmitic acid (Figures 3, 4). However, in dEoL-1 cells, palmitic acid treatment also induced a significant increase in the Th2 cytokine transcripts (*IL5* and *IL13*) and secretion of IL-16 (Figures 3, 4), which regulate adaptive immune responses and support eosinophil recruitment and activation (Luna-Gomes et al., 2013; Richmond et al., 2014). Moreover, the dEoL-1 cells showed increased expression of GATA-1 and GATA-3, transcription factors regulating maturation of functional eosinophils, upon stimulation with palmitic acid (Figure 5; Yamaguchi et al., 1998; Zhang et al., 1998). Lipid breakdown and fatty acid oxidation provides an energy source that is used by immune cells (O’Sullivan et al., 2014), and fatty acids are critical for neutrophil differentiation because they support mitochondrial respiration (Riffelmacher et al., 2017). CB eosinophils showed expression of genes encoding long-chain fatty acid transport proteins (*SLC27A1*, *SLC27A2*, *SLC27A3*, *SLC27A4*, and *SLC27A5*) (Kazantzis and Stahl, 2012); however, mRNA expression of *SLC27A2* and *SLC27A5* was significantly decreased in EoL-1 with butyrate-induced differentiation (Supplementary Figure S8), suggesting the limited role of signals transduced by fatty acid transport proteins in eosinophils. As the role of fatty acids in the functional responses of eosinophils remains largely unknown, further research is required to identify the contribution of fatty acids in the maturation of eosinophil-lineage differentiated cells or in TLR-mediated signaling. Considering that the expression of inflammatory cytokines was not significantly increased by butyrate-induced EoL-1 cell differentiation alone (Figure 3), it is plausible that eosinophils sense the pathogen-associated molecular patterns or damage-associated molecular patterns of TLR4, and provide immune mediators for their microenvironment in a ligand-dependent manner. In this study, we used EoL-1 cells as alternative to circulating eosinophils, however, functional aspects of EoL-1 cells or blood eosinophils may differ from tissue-resident eosinophils. Therefore, fatty acid-induced functional changes of tissue-resident eosinophils need to be investigated.

Obesity induces chronic inflammation in the adipose tissue with an increased infiltration of macrophages, preferentially the pro-inflammatory M1 phenotype (Lumeng et al., 2007). Under steady state, adipose tissue eosinophils support the polarization of anti-inflammatory M2 macrophages (Wu et al., 2011). Although both LPS and palmitic acid induced inflammatory changes in dEoL-1 cells, the cytokine profile induced by LPS shifted to pro-inflammatory compared to palmitic acid treatment (Figures 3, 4). Additionally, expression of transcription factors associated with eosinophil development was prominently increased in dEoL-1 cells treated with palmitic acid compared to LPS (Figure 5). Thus, we suggest that eosinophils influence the polarization of macrophages by modulating microenvironments sensed by TLR4. This idea was supported by the significantly increased expression of M1 macrophage representative markers such as *IL1B*, *TNF*, *IDO* in M0 differentiated THP-1 cells treated with CM collected from LPS-stimulated dEoL-1 cells, and by the up-regulated M2 macrophage markers *MAF* and *FCER2* by treatment with CM collected from palmitic acid-stimulated dEoL-1 cells (Figure 6).

The stored fat in lean individuals is the major organ for free fatty acid flux and host eosinophils that secrete M2 polarizing cytokines, such as IL-4, IL-5, and IL-13 (Mittendorfer, 2011; Wu et al., 2011). However, obese adipose tissue is characterized by the infiltration of monocytes and increased expression of pro-inflammatory cytokines, such as TNF-α, IL-6, and IL-1β, which promote the differentiation of M1 macrophages (Castoldi et al., 2015). In line with this, we previously reported significantly increased infiltration of macrophages in the obese adipose tissue of eosinophil-deficient mice, with a marked increase in IFN-γ and decrease in IL-4 and IL-13 levels when compared to those in the WT control (Lee et al., 2018). Accordingly, eosinophils and M2 macrophages were significantly decreased in the adipose tissue, despite an increase in total macrophages in case of obesity induced by the HFD (Figure 7 and Supplementary Figure S6). Tissue levels of eosinophils can be decreased by inflammatory signals within tissues (Zimmermann and Rothenberg, 2015), and obesity induces a low-grade chronic inflammation in the adipose tissue with decrease of eosinophils (Weisberg et al., 2003; Wu et al., 2011). Considering that excessive adipocyte enlargement due to obesity leads to adipocyte dysfunction and decrease of fatty acid uptake (McQuaid et al., 2011; Mittendorfer, 2011), we propose that decreased eosinophils and palmitic acid signals sensed by eosinophils might account for the decrease of M2 macrophages in the adipose tissue of obese mice at least in part. This idea was further supported by the insignificant changes of eosinophils and M2 macrophages in the small intestine of HFD-fed mice (Figure 7B and Supplementary Figure S6), where most of tissue eosinophils are constitutively present regardless of obesity (Jung and Rothenberg, 2014; Lee et al., 2018).

Eosinophils serve as a source of a number of cytokines and growth factors and can modulate various immune responses through an array of orchestrated interactions with other immune cells. Our data demonstrate that differentiated human eosinophil cell line and mature eosinophils derived from CB express TLR4, and that stimulating an eosinophil cell line with TL4 ligands of LPS or palmitic acid supports macrophage polarization toward M1 or M2, respectively. Based on our findings, we propose that microenvironment signals sensed by TLR4 in eosinophils modulate both inflammatory and anti-inflammatory immune responses, and that reinforcing the regulatory effect of eosinophils could promote metabolic homeostasis. Studying the detailed mechanism underlying interactions between eosinophils and other immune cells may provide new insights that could contribute to achieving better control of diseases associated with eosinophil dysfunction.

## Data Availability Statement

The datasets generated for this study can be found in the Gene Expression Omnibus database/GSE54667.

## Ethics Statement

The studies involving human participants were reviewed and approved by the ethics committees of Hanyang University. The patients/participants provided their written informed consent to participate in this study. The animal study was reviewed and approved by the Center of Animal Care and Use of Lee Gil Ya Cancer and Diabetes Institute, Gachon University.

## Author Contributions

JY and H-NU researched and analyzed the data, and wrote the manuscript. JJ researched and analyzed the data, and reviewed the manuscript. Y-AB, W-JP, HK, M-SY and IC analyzed the data, reviewed the manuscript, and contributed to the discussion. YJ designed and supervised the studies, analyzed the data, and wrote and reviewed the manuscript.

## Conflict of Interest

The authors declare that the research was conducted in the absence of any commercial or financial relationships that could be construed as a potential conflict of interest.

## Supplementary Material

FIGURE S1 EoL-1 cell differentiation by butyrate treatment. **(A)** mRNA expressions of *CCR3*, *ECP*, and *EDN* in EoL-1 cells differentiated with 0.5 mM butyrate for 2 days. Data are presented as mean ± SD. ^∗^*p* < 0.05 (Mann-Whitney U test). **(B)** Representative flow cytometry plot and mean fluorescence intensity (MFI) of IL-5Rα in butyrate-differentiated EoL-1 (dEoL-1) cells. Isotype controls are in grey. Data are presented as mean ± SD. ^∗∗∗^*p* < 0.001 (Student’s *t*-test).

FIGURE S2 Eosinophils differentiated from human cord blood CD34^+^ cells. Representative photos of developing cord blood eosinophils stained with Diff-Quick solution, which contain anionic Eosin Y and cationic thiazine, and highlight the cytoplasm of eosinophils. Mature eosinophils with pinkish cytoplasmic granules and fragmented nuclei were prominent in the culture on day 24. Original magnification × 100.

FIGURE S3 Expression of inflammatory cytokines in dEoL-1 cells stimulated with TLR4 ligands. mRNA expression of *IL1B*, *IL6*, and *TNF* was analyzed by real-time PCR in dEoL-1 cells stimulated with indicated concentrations of lipopolysaccharide (LPS) or palmitic acid. Data are presented as mean ± SD. ^∗^*p* < 0.05, ^∗∗^*p* < 0.01 (Kruskal-Wallis test).

FIGURE S4 Quantification of the density of each spot detected via the protein array analysis of cytokines in the culture supernatants of EoL-1 cells. Chemiluminescence signal produced in proportion to the amount of cytokines was detected using an ImageQuant LAS 4000 biomolecular imager and the intensity of signal was quantified with the Dot blot Analyzer tool of ImageJ software. Data are presented as mean ± SD.

FIGURE S5 THP-1 cells polarized to either M1 or M2 macrophage phenotype. Human monocyte THP-1 cell line was subjected to the following activation treatments; no stimulation control; 100 ng/mL of PMA (Sigma-Aldrich, St. Louis, MO, United States) for 24 h (M0 polarization); pretreatment with PMA for 24h, followed by 10 pg/mL of LPS (Sigma-Aldrich) and 20 ng/mL of IFN-γ (Peprotech, Rocky Hill, NJ, United States) for 24 h (M1 polarization); pretreatment with PMA for 24h, followed by 30 ng/mL of IL-4 (Peprotech) and 20 ng/mL of IL-13 (Peprotech) for 24 h (M2 polarization). Expression of M1 (*IL1B, TNF, IDO*) or M2-representative markers (*MAF, FCER2, CCL17*) in untreated control and polarized THP-1 cells (M0, M1, M2) was analyzed using real-time PCR. Data are presented as mean ± SD. ^∗^*p* < 0.05, ^∗∗^*p* < 0.01, ^∗∗∗^*p* < 0.001 (Kruskal-Wallis test).

FIGURE S6 Gating strategy for eosinophils and macrophages isolated from the adipose tissue and small intestine of mice fed with chow or high fat diet (HFD). **(A)** Flow cytometry analysis of CD45^+^CCR3^+^SiglecF^+^ eosinophils and **(B)** CD45^+^F4/80^+^ macrophages, CD45^+^F4/80^+^CD11c^+^CD206^–^ M1 macrophages, and CD45^+^F4/80^+^CD11c^–^CD206^+^ M2 macrophages in the adipose tissue and small intestine of mice on a chow or HFD. Representative dot plots are shown.

FIGURE S7 Viability of cells isolated from adipose tissue and small intestine of mice fed with chow or the high-fat diet (HFD). Viability of the isolated leukocytes was determined by flow cytometry analysis of 7-amino-actinomycin D (7-AAD). **(A)** CD45^+^7-AAD^–^ viable leukocytes and CD45^+^7-AAD^+^ non-viable leukocytes in the adipose tissue and small intestine of mice fed the chow diet or HFD. Representative dot plots are shown. **(B)** Frequency of CD45^+^7-AAD^–^ viable leukocytes. Data are presented as mean ± SD (Mann-Whitney U test).

FIGURE S8 Expression of fatty acid transporters in EoL-1 cells and mature eosinophils differentiated from human cord blood (CB) CD34^+^ cells. **(A)** mRNA expression for *SLC27A2* and *SLC27A5* in undifferentiated EoL-1 cells and butyrate-differentiated EoL-1 cells were analyzed using real-time PCR. Data are presented as mean ± SD; ^∗∗∗^*p* < 0.001 (Mann-Whitney U test for *SLC27A2*, Student’s *t*-test for *SLC27A5*). **(B)** Expression of genes associated with fatty acid transport in CB-differentiated eosinophils. The expression was analyzed by RNA-sequencing and the values represent fragment per kilobase per million map reads (FPKM) of genes. Dashed line indicates FPKM = 1. (*n* = 3 per group). Data are presented as mean ± SD.

TABLE S1 Primer sequences for real-time PCR.

IMAGE S1 riginal blot image 1. Butyrate.

IMAGE S2 riginal blot image 2. Butyrate+LPS.

IMAGE S3 riginal blot image 3. Butyrate+Palmitic acid.

IMAGE S4 riginal blot image 4. β-actin.

IMAGE S5 riginal blot image 5. GATA-1.

IMAGE S6 riginal blot image 6. GATA-3.

IMAGE S7 riginal blot image 7. p38.

IMAGE S8 riginal blot image 8. Phospho p38.

IMAGE S9 riginal blot image 9. Phospho p44/42.

IMAGE S10 riginal blot image 10. p44/42.
