# Corrigendum: Eosinophil Activation by Toll-Like Receptor 4 Ligands Regulates Macrophage Polarization

**DOI:** 10.3389/fcell.2020.00003

**Published:** 2020-01-28

**Authors:** Jiyoung Yoon, Han-Na Um, Jinsun Jang, Young-An Bae, Woo-Jae Park, Hee Joo Kim, Mee-Sup Yoon, Il Yup Chung, YunJae Jung

**Affiliations:** ^1^Department of Microbiology, College of Medicine, Gachon University, Incheon, South Korea; ^2^Department of Health Sciences and Technology, GAIHST, Gachon University, Incheon, South Korea; ^3^Department of Dermatology, Gachon Gil Medical Center, College of Medicine, Gachon University, Incheon, South Korea; ^4^Department of Biochemistry, College of Medicine, Gachon University, Incheon, South Korea; ^5^Department of Molecular Medicine, College of Medicine, Gachon University, Incheon, South Korea; ^6^Department of Bionano Technology, Hanyang University, Ansan, South Korea

**Keywords:** eosinophils, EoL-1 cells, TLR4, THP-1 cells, macrophage polarization

An author name was incorrectly spelled as “Ip Yup Chung.” The correct spelling is “Il Yup Chung.”

The authors apologize for this error and state that this does not change the scientific conclusions of the article in any way. The original article has been updated.

