# Supplementary material for: Eosinophil Activation by Toll-Like Receptor 4 Ligands Regulates Macrophage Polarization
Source: Front Cell Dev Biol. 2019 Dec 20;7:329. doi: 10.3389/fcell.2019.00329 (PMC6933835; doi:10.3389/fcell.2019.00329)
Supplement: FIGURE S1 — EoL-1 cell differentiation by butyrate treatment. (A) mRNA expressions of CCR3, ECP, and EDN in EoL-1 cells differentiated with 0.5 mM butyrate for 2 days. Data are presented as mean ± SD. ∗p < 0.05 (Mann-Whitney U test). (B) Representative flow cytometry plot and mean fluorescence intensity (MFI) of IL-5Rα in butyrate-differentiated EoL-1 (dEoL-1) cells. Isotype controls are in grey. Data are presented as mean ± SD. ∗∗∗p < 0.001 (Student’s t-test). [file Data_Sheet_1.docx]

Supplementary Material

# Supplementary Figures and Table

## Supplementary Figures

**
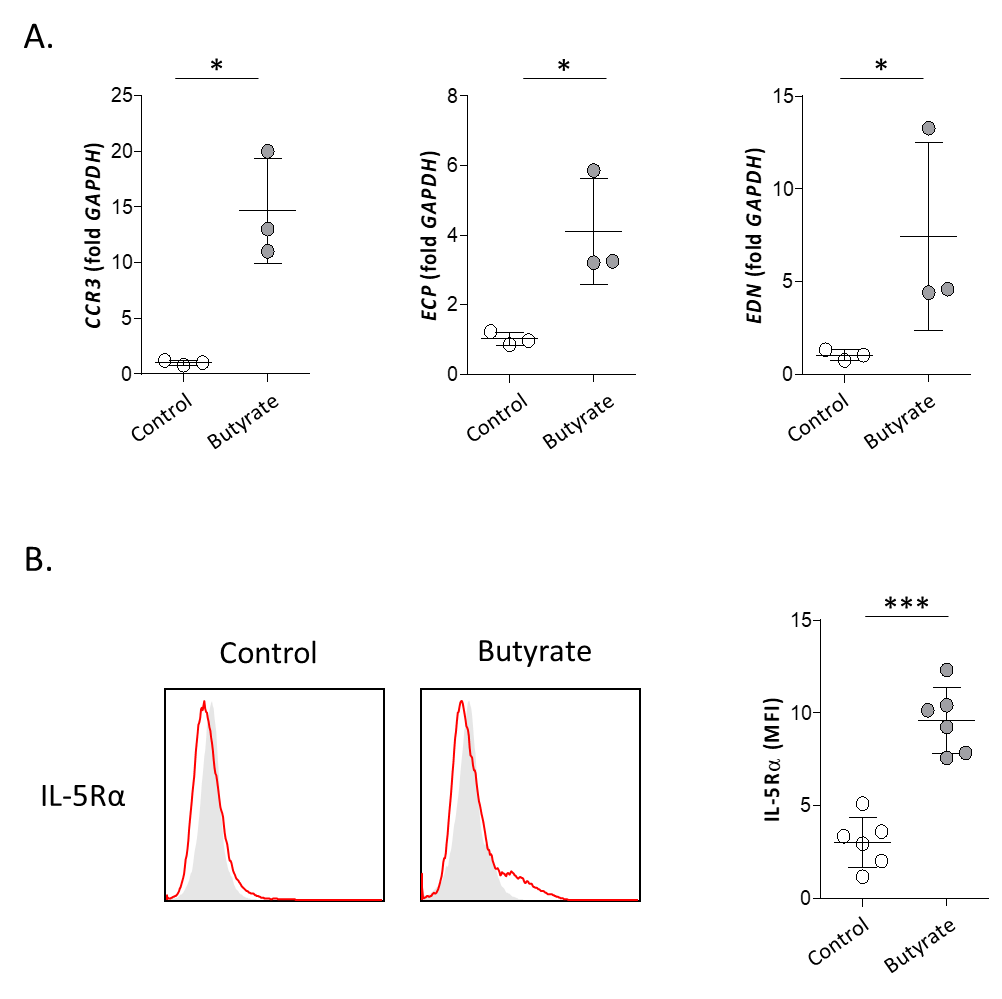
**

**Figure S1. EoL-1 cell differentiation by butyrate treatment.** (A) mRNA expressions of *CCR3, ECP,* and *EDN* in EoL-1 cells differentiated with 0.5 mM butyrate for 2 days. Data are presented as mean ± SD. **p* < 0.05 (Mann-Whitney U test). (B) Representative flow cytometry plot and mean fluorescence intensity (MFI) of IL-5Rα in butyrate-differentiated EoL-1 (dEoL-1) cells. Isotype controls are in grey. Data are presented as mean ± SD. ****p <* 0.001 (Student’s *t*-test).

**
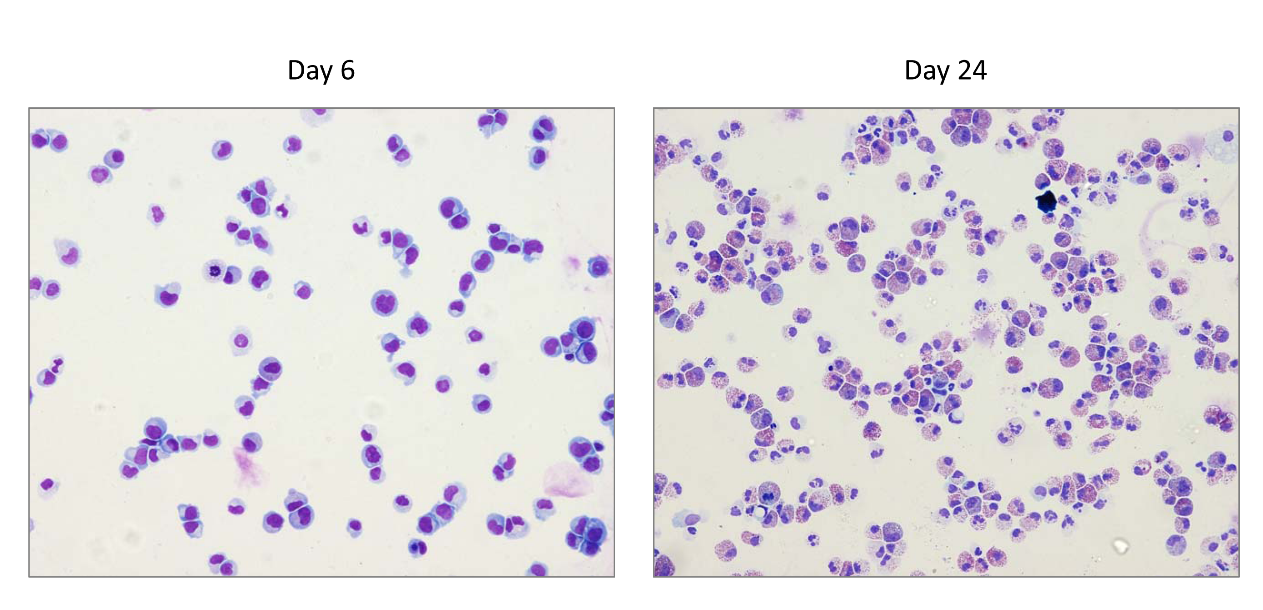
**

**Figure S2. Eosinophils differentiated from human cord blood CD34^+^ cells.** Representative photos of developing cord blood eosinophils stained with Diff-Quick solution, which contain anionic Eosin Y and cationic thiazine, and highlight the cytoplasm of eosinophils. Mature eosinophils with pinkish cytoplasmic granules and fragmented nuclei were prominent in the culture on day 24. Original magnificationⅹ100.

**
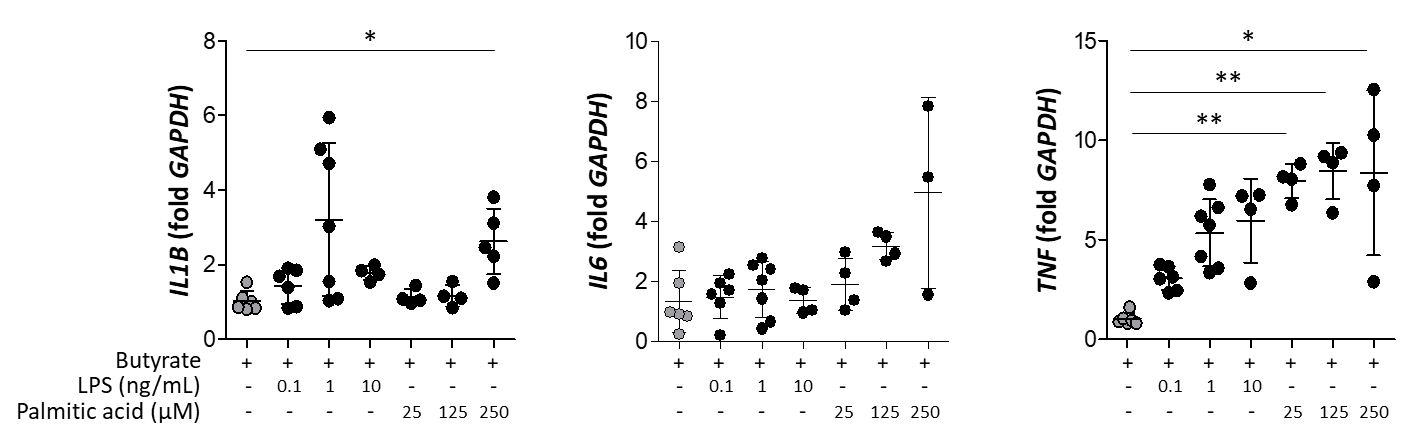
**

**Figure S3. Expression of inflammatory cytokines in dEoL-1 cells stimulated with TLR4 ligands.** mRNA expression of *IL1B*, *IL6*, and *TNF* was analyzed by real-time PCR in dEoL-1 cells stimulated with indicated concentrations of lipopolysaccharide (LPS) or palmitic acid. Data are presented as mean ± SD. **p* < 0.05, ***p* < 0.01 (Kruskal-Wallis test).

**
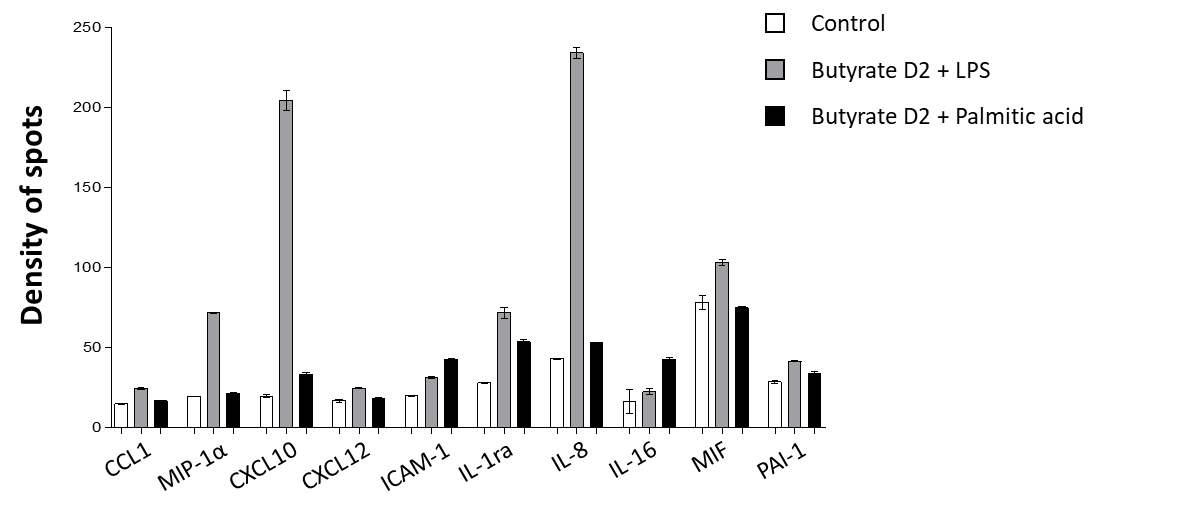
**

**Figure S4. Quantification of the density of each spot detected via the protein array analysis of cytokines in the culture supernatants of EoL-1 cells.** Chemiluminescence signal produced in proportion to the amount of cytokines was detected using an ImageQuant LAS 4000 biomolecular imager and the intensity of signal was quantified with the [Dot blot](https://www.sciencedirect.com/topics/agricultural-and-biological-sciences/dot-blot) Analyzer tool of ImageJ software. Data are presented as mean ± SD.

**
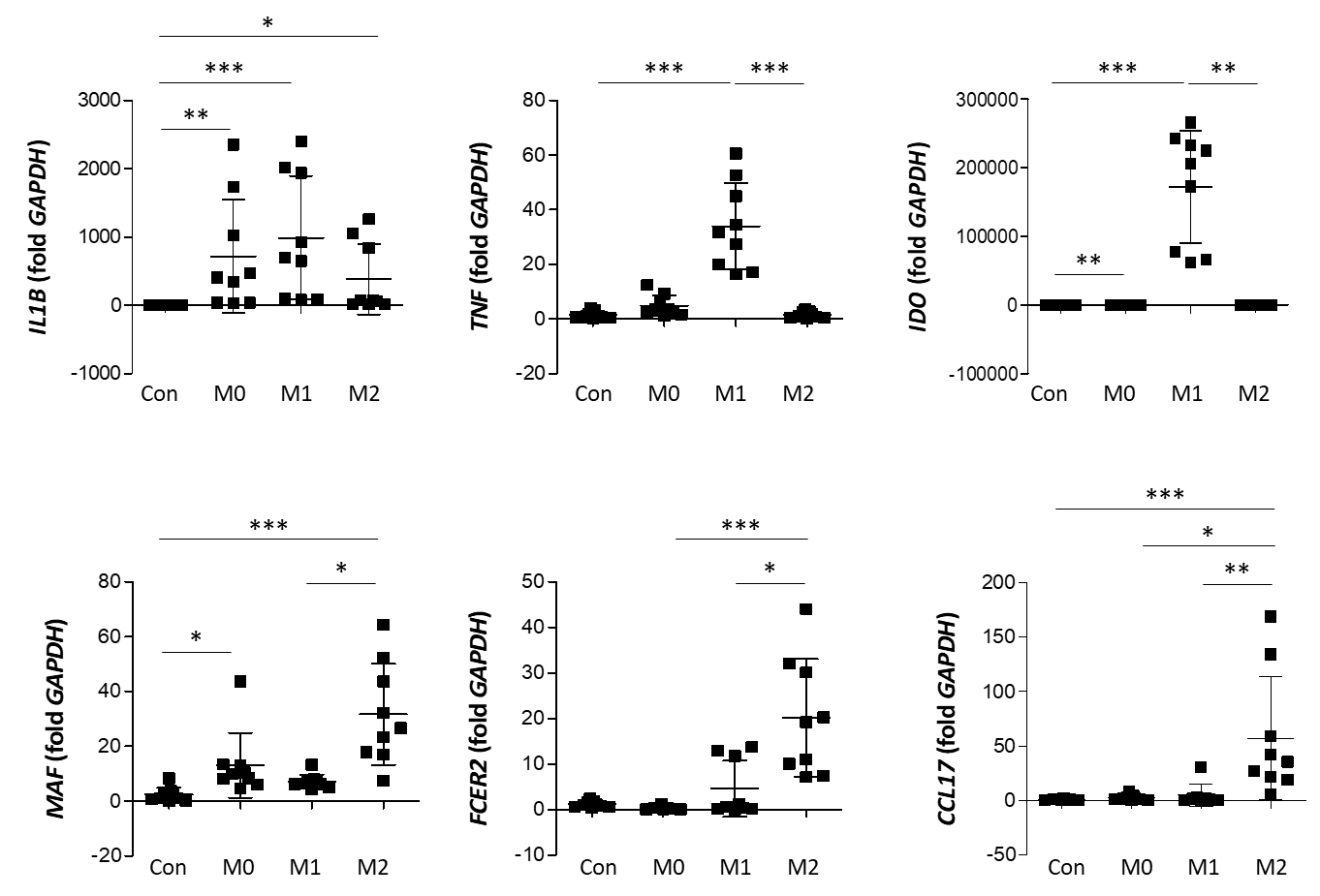
**

**Figure S5. THP-1 cells polarized to either M1 or M2 macrophage phenotype.** Human monocyte THP-1 cell line was subjected to the following activation treatments; no stimulation control; 100 ng/mL of PMA (Sigma-Aldrich, St. Louis, MO, USA) for 24 h (M0 polarization); pretreatment with PMA for 24h, followed by 10 pg/mL of LPS (Sigma-Aldrich) and 20 ng/mL of IFN-γ (Peprotech, Rocky Hill, NJ, USA) for 24 h (M1 polarization); pretreatment with PMA for 24h, followed by 30 ng/mL of IL-4 (Peprotech) and 20 ng/mL of IL-13 (Peprotech) for 24 h (M2 polarization). Expression of M1 (*IL1B, TNF, IDO*) or M2-representative markers (*MAF, FCER2, CCL17*) in untreated control and polarized THP-1 cells (M0, M1, M2) was analyzed using real-time PCR. Data are presented as mean ± SD. **p <* 0.05, ***p* < 0.01, ****p <* 0.001 (Kruskal-Wallis test).

**
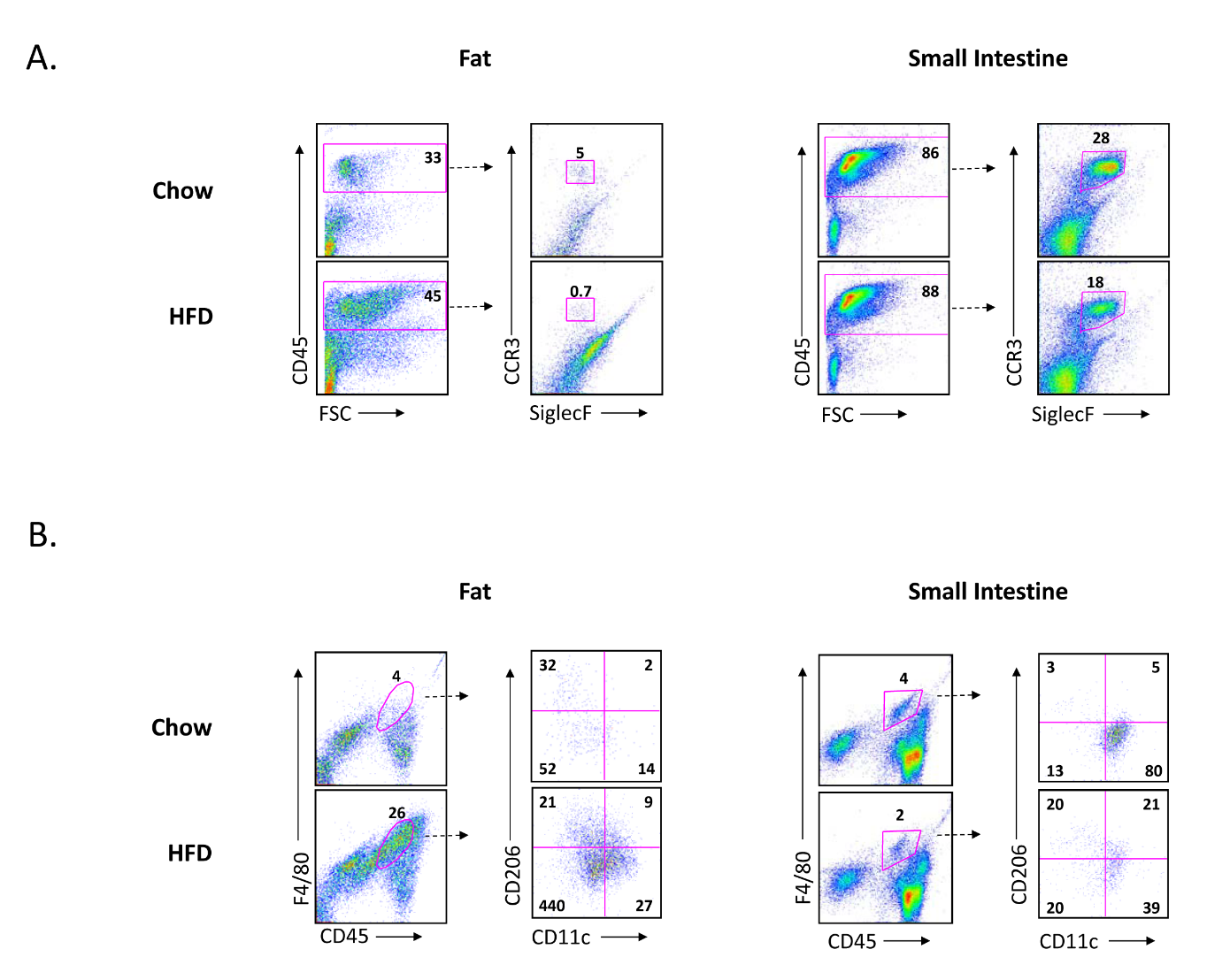
**

**Figure S6. Gating strategy for eosinophils and macrophages isolated from the adipose tissue and small intestine of mice fed with chow or high fat diet (HFD).** (A) Flow cytometry analysis of CD45^+^CCR3^+^SiglecF^+^ eosinophils and (B) CD45^+^F4/80^+^ macrophages, CD45^+^F4/80^+^CD11c^+^CD206^-^ M1 macrophages, and CD45^+^F4/80^+^CD11c^-^CD206^+^ M2 macrophages in the adipose tissue and small intestine of mice on a chow or HFD. Representative dot plots are shown.

**
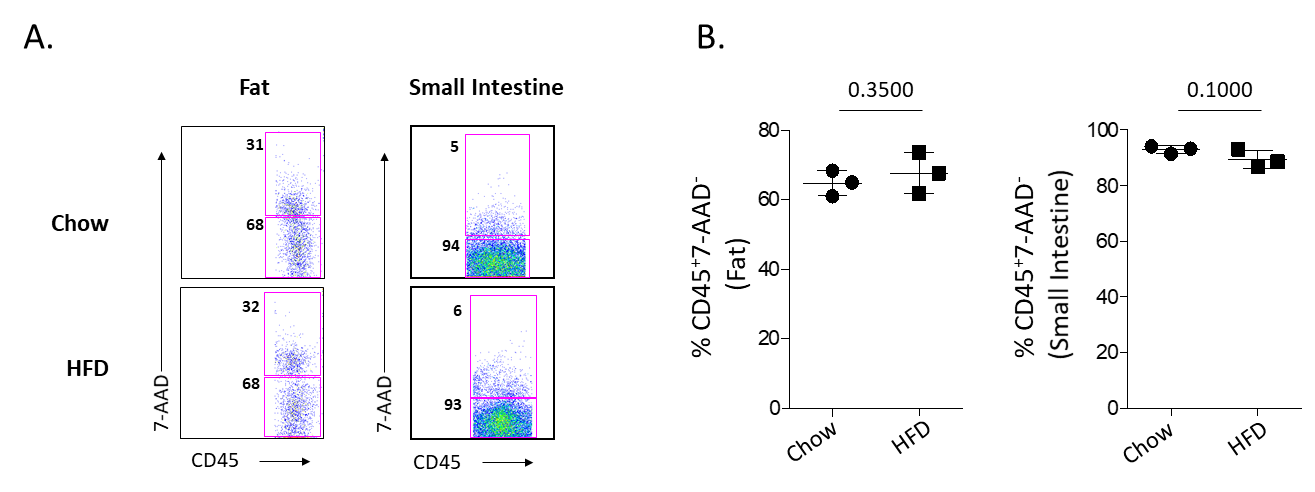
**

**Figure S7. Viability of cells isolated from adipose tissue and small intestine of mice fed with chow or the high-fat diet (HFD).** Viability of the isolated leukocytes was determined by flow cytometry analysis of 7-amino-actinomycin D (7-AAD). (A) CD45^+^7-AAD^-^ viable leukocytes and CD45^+^7-AAD^+^ non-viable leukocytes in the adipose tissue and small intestine of mice fed the chow diet or HFD. Representative dot plots are shown. (B) Frequency of CD45^+^7-AAD^-^ viable leukocytes. Data are presented as mean ± SD (Mann-Whitney U test).

**
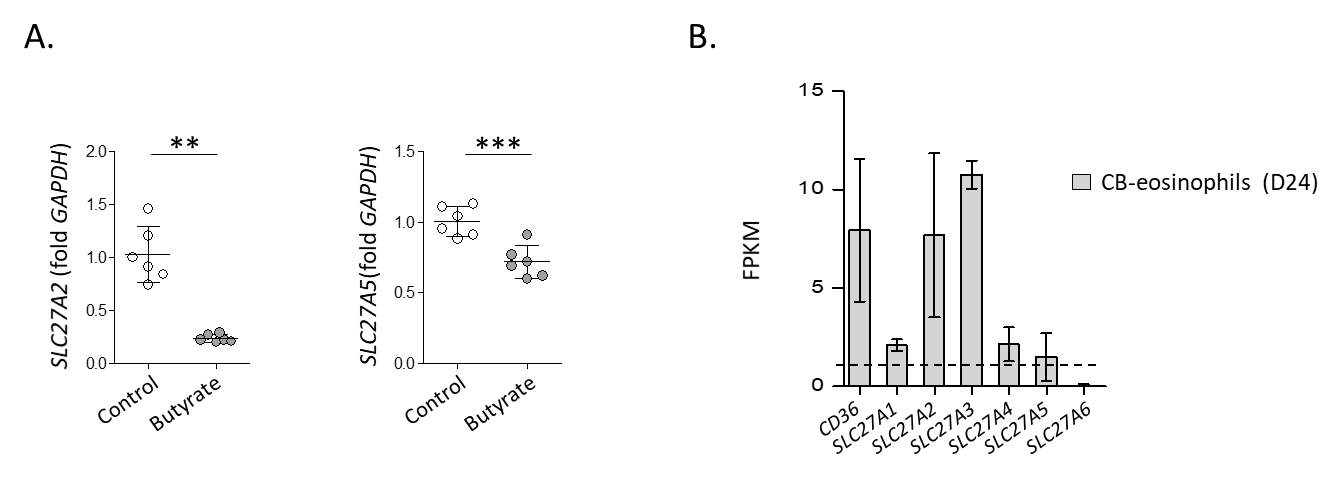
**

**Figure S8. Expression of fatty acid transporters in EoL-1 cells and mature eosinophils differentiated from human cord blood (CB) CD34^+^ cells.** (A) mRNA expression for *SLC27A2* and *SLC27A5* in undifferentiated EoL-1 cells and butyrate-differentiated EoL-1 cells were analyzed using real-time PCR. Data are presented as mean ± SD; ****p* < 0.001 (Mann-Whitney U test for *SLC27A2*, Student’s *t*-test for *SLC27A5*). (B) Expression of genes associated with fatty acid transport in CB-differentiated eosinophils. The expression was analyzed by RNA-sequencing and the values represent fragment per kilobase per million map reads (FPKM) of genes. Dashed line indicates FPKM = 1. (*n* =3 per group). Data are presented as mean ± SD

## Supplementary Table

## Table S1. Primer sequences for real-time PCR

| Target gene | Primer sequence |  |  |
| --- | --- | --- | --- |
| *TLR1* | Forward: 5′-CTG TAG CAA ATC TGG AAC TAT C-3′  Reverse: 5′-GTT GTT TCA ATG TTG TTT AAG GT-3′ | | |
| *TLR2* | Forward: 5′-CAT AAG CGG GAC TTC ATT C-3′  Reverse: 5′-CAG CAT CAT TGT TCT CAT CA-3′ | | |
| *TLR3* | Forward: 5′-CAC CAC CAG CAA TAC AAC-3′  Reverse: 5′-AAG CCA AGC AAA GGA ATC-3′ | | |
| *TLR4* | Forward: 5′-CAG GTT CTT GAT TAC AGT CTC-3′  Reverse: 5′-GCA CAT TCC ATT CGT TCA-3′ | | |
| *TLR6* | Forward: 5′-TCG CTA TCC AAG TGA ACA T-3′  Reverse: 5′-GCA TTT CCA AGT CGT TTC TA-3′ | | |
| *TLR7* | Forward: 5′-GCT CTG TGG GAG TTC TGT CC-3′  Reverse: 5′-ACC GTT TCC TTG AAC ACC TG-3′ | | |
| *TLR8* | Forward: 5′-CTA CCC TCT GGC TTT CTT TC-3′  Reverse: 5′-GTG CGG ATT TGT TGA TTG TT-3′ | | |
| *TLR9* | Forward: 5′-TGG AGT ATC TGC TGT TGT-3′  Reverse: 5′-TGA AGG TAT CGG GAT GTA G-3′ | | |
| *IL1B* | Forward: 5′-ATG ATG GCT TAT TAC AGT GGC AA-3′  Reverse: 5′-GTC GGA GAT TCG TAG CTG GA-3′ | | |
| *IL6* | Forward: 5′-GAC AGC CAC TCA CCT CTT CAG A-3′  Reverse: 5′-GTG CCT CTT TGC TGC TTT CAC-3′ | | |
| *TNF* | Forward: 5′-CCT GTG AGG AGG ACG AAC AT-3′  Reverse: 5′-GGT TGA GGG TGT CTG AAG GA-3′ | | |
| *IL5* | Forward: 5′-TCT ACT CAT CGA ACT CTG CTG A-3′  Reverse: 5′-CCC TTG CAC AGT TTG ACT CTC-3′ | | |
| *IL13* | Forward: 5′-TGT TTG TCA CCG TTG GGG AT-3′  Reverse: 5′-TGA GTC TCT GAA CCC TTG GC-3′ | | |
| *IFNG* | Forward: 5′-TCG GTA ACT GAC TTG AAT GTC CA-3′  Reverse: 5′-TCG CTT CCC TGT TTT AGC TGC-3′ | | |
| *IDO1* | Forward: 5′-CGA GAA AGA GTT GAG AAG TT-3′  Reverse: 5′-TGG AGA GTT GGC AGT AAG-3′ | | |
| *MAF* | Forward: 5′-CCC TCC CTC TCC TCC AGT C-3′  Reverse: 5′-ATG CTT CTC GCC TCC TCT TCT-3′ | |  |
| *FCER2* | Forward: 5′-GCG TGG GAC TCA GAT CGT G-3′  Reverse: 5′-GCT GTT TTA GAC TCT GTG TGG TG-3′ | |  |
| *CCL17* | Forward: 5′-CGG ACC CCA ACA ACA AGA GA-3′  Reverse: 5′-CTC CCT CAC TGT GGC TCT TC-3′ | |  |
| *SLC27A2* | Forward: 5′-CAT CAG CGC ATA TGG TAT GG-3′  Reverse: 5′-TGC AGC ACT GTG GTA AAA GG-3′ | |  |
| *SLC27A5* | Forward: 5′-GAT CGT GGT TGA CCC TCT GT-3′  Reverse: 5′-CTC CAG GTT CCC TCA CAC AC-3′ | |  |
| *GAPDH* | Forward: 5′-CTG GGC TAC ACT GAG CAC C-3′  Reverse: 5′-AAG TGG TCG TTG AGG GCA ATG-3′ | |  |
