# Supplementary figures and images for: Eosinophil Activation by Toll-Like Receptor 4 Ligands Regulates Macrophage Polarization
Source: Front Cell Dev Biol. 2019 Dec 20;7:329. doi: 10.3389/fcell.2019.00329 (PMC6933835; doi:10.3389/fcell.2019.00329)

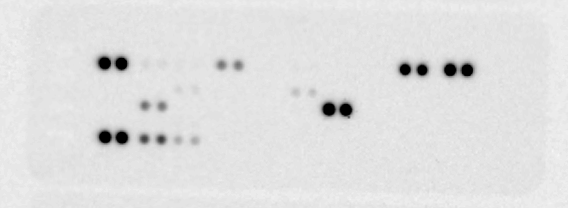

Supplement: IMAGE S2 — riginal blot image 2. Butyrate+LPS. [file Image_2.TIF]

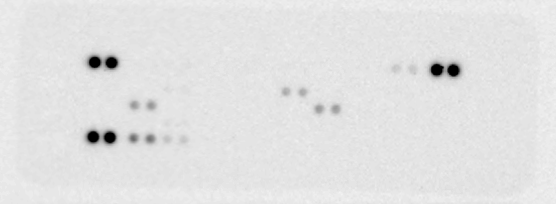

Supplement: IMAGE S3 — riginal blot image 3. Butyrate+Palmitic acid. [file Image_3.TIF]

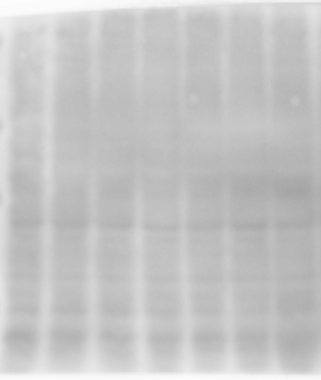

Supplement: IMAGE S5 — riginal blot image 5. GATA-1. [file Image_5.TIF]

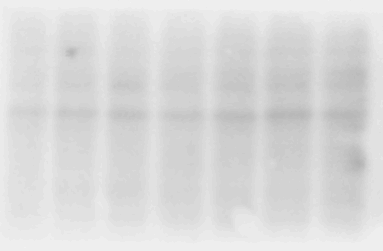

Supplement: IMAGE S6 — riginal blot image 6. GATA-3. [file Image_6.TIF]

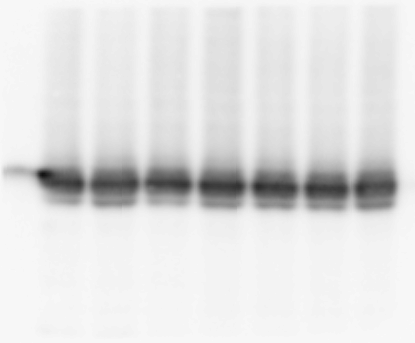

Supplement: IMAGE S7 — riginal blot image 7. p38. [file Image_7.TIF]

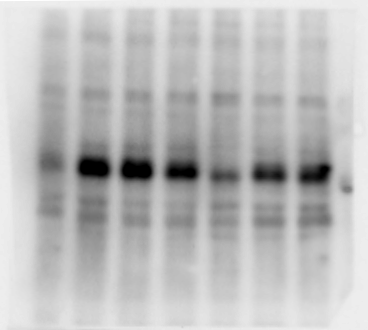

Supplement: IMAGE S8 — riginal blot image 8. Phospho p38. [file Image_8.TIF]

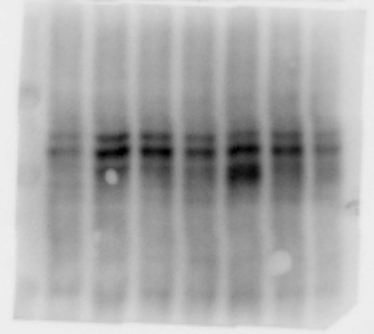

Supplement: IMAGE S9 — riginal blot image 9. Phospho p44/42. [file Image_9.TIF]
